# Enhanced immune reconstitution with albuvirtide in HIV-infected immunological non-responders

**DOI:** 10.3389/fcimb.2024.1397743

**Published:** 2024-06-21

**Authors:** Lina Fan, Yue Hu, Rui Li, Jiaqi Ding, Yuantao Liu, Shuchang Yu, Min Hu, Rui Su, Yangyang Li, AiPing Yu, Dong Xie, Qingxia Zhao, Ping Ma

**Affiliations:** ^1^ Department of Infectious Diseases, Tianjin Second People's Hospital, Tianjin, China; ^2^ Tianjin Institute of Hepatology, Tianjin Second People’s Hospital, Tianjin, China; ^3^ Tianjin Medical University, Tianjin, China; ^4^ Medical Affairs Department, Frontier Biotechnologies Inc, Nanjing, Jiangsu, China; ^5^ Department of Pathology, Tianjin Second People's Hospital, Tianjin, China; ^6^ Department of Infectious Diseases, Henan Infectious Diseases Hospital, Zhengzhou, Henan, China; ^7^ Tianjin Association of STD/AIDS Prevention and Control, Tianjin, China

**Keywords:** HIV, incomplete immune reconstitution, albuvirtide, intensification, CD4+ T lymphocyte

## Abstract

**Background:**

Incomplete immune recovery in people living with HIV/AIDS (PLWHA) remains an important clinical challenge with the lack of an effective strategy currently available to restore their T-cell immune response. This study aimed to evaluate the effect of Albuvirtide (ABT) on immune recovery in immunological non-responders (INRs) and attempted to explore potential mechanisms of ABT on the functionality of immune cells.

**Methods:**

In this prospective, open-label, controlled clinical study, participants with incomplete immune reconstitution (continuous ART over 5 years and CD4^+^T lymphocyte absolute count of <500 cells/µl or ART for 2–5 years and CD4^+^T cell count of <200 cells/µl with undetectable viral load) were received intensive treatment with ABT or maintained on the original ART regimen at a ratio of 1:1. Immune response and safety were examined within 24 weeks. In the cytological study, T subsets, cell apoptosis and cell autophagy were analyzed using immunofluorescence staining and flow cytometry from 25 blood specimens.

**Results:**

Both groups (n=25 each) were comparable in age, gender, and ART duration. At week 12, CD4^+^T cell count increased significantly in the intensive ABT group compared with control group (the change from baseline in CD4^+^T cell count: 45 vs. -5 cells/µL, *p*<0.001). After ABT discontinuation, CD4^+^T cell counts remained significantly higher in the intensive ABT group at week 24 (55 vs. -5 cells/µL, *p*=0.012). In laboratory analysis, naïve CD4^+^ T cell amounts were lowest among participants with unsatisfactory immune response (uIR) to ABT (*p*=0.001). The proportion of caspase 3^+^CD45RA^+^CD31^+^CD4^+^ T cells was significantly lower in participants with satisfactory immune response (sIR) to ABT (*p*<0.05).

**Conclusion:**

Significant CD4^+^T cell count increase suggests ABT enhances immune function in INRs which may be attributed to its antiviral properties as well as its ability to increase thymic cell output and decrease cell apoptosis.

## Introduction

Antiretroviral therapy (ART) is highly successful in the treatment of people living with HIV/AIDS (PLWHA). However, some PLWHA do not achieve immune reconstitution and are referred to as immunological non-responders (INRs). INRs are defined as PLWHA with CD4^+^T cell counts below 350 cells/µL (Rb-Silva et al., 2019), despite full virologic suppression (generally HIV RNA <50 copies/mL) for at least two years. In China, approximately 20–30% of PLWHA are INRs (Medicine A-ABoCAoC, 2020).

Poor immune recovery leads to AIDS progression, increased incidence rates of comorbidities (e.g., cancer and opportunistic infections) and long-term morbidity (Engsig et al., 2014; Takuva et al., 2014; Noiman et al., 2022). Potential contributing factors of INRs include reduced thymic function, persistent viral replication, abnormal immune activation, and dysregulated cytokines (Yang et al., 2020). Additionally, thymic exhaustion is one of the major factors affecting INRs.

Currently, therapeutic approaches to restore immune response for INRs are undefined. Several interventions such as (5R)-5-hydroxytriptolide and IL-7 have been studied and reported to have promising effect on immune reconstitution (Cao et al., 2023). Also, applying maraviroc and raltegravir intensified ART has been studied for its effect on immune activation (Corbeau and Reynes, 2011). However, intensification regimens did not report consistent efficacy or had limited evidence strength (Yang et al., 2020). There remains a need to further explore intensification regimens for immune reconstitution in individuals with INRs.

ABT, a long-acting fusion inhibitor, has been widely used in China since its launched. Previous investigations have predominantly focused on the reduction of viral load and the increase in CD4^+^T cell count in both treatment-naïve and treatment-experienced PLWHA. In the phase III, open-label, randomized, multicenter, parallel-group, non-inferiority study, ABT plus ritonavir-boosted lopinavir (LPV/r) significantly elevates CD4^+^T cell amounts by 50 cells/µL in four weeks (Zhang et al., 2016; Chen et al., 2022). Clinical evidence suggests that ABT is promising in offering additional immune recovery for HIV-infected individuals with incomplete immune reconstitution (Zhang et al., 2016; Su et al., 2020; Chen et al., 2022; Su et al., 2022; Tang et al., 2023). Previous *vitro* and *ex vivo* assays demonstrated that fusion inhibitors, including T20 and C34, who have similar amino acid sequences with ABT can prevent Env-mediated cellular autophagy and reduce thymocyte apoptosis (Meissner et al., 2006; Doitsh et al., 2014). In this study, we aim to broaden the scope of ABT to investigate its potential in immune reconstitution in INRs after long-term ART and an exploratory study was performed to identify how ABT mediate T cell subsets to enhance immune recovery.

## Methods

### Study design and participants in the clinical study

This prospective, open-label, controlled study was performed at Tianjin Second People’s Hospital in Tianjin, China, and had approval from the Ethics Committee of the above hospital (Ethics Committee approval code: 2021–35).

From November 2021 to August 2023, fifty participants were assigned to and received treatment either in the ABT or control group at a 1:1 ratio. In the control group, the patients continued their original ART regimens if no modifications were required for HIV care. In the ABT group, the participants received additional ABT at 320 mg IV once daily on days 1, 2, and 3, and weekly from day 8 onwards till week 12. Follow-up was performed at weeks 12 and 24.

Eligible participants were individuals aged 18–65 diagnosed with HIV infection, under antiretroviral therapy (ART) for at least 5 years with CD4^+^T cell counts below 500 cells/µL, or under ART for 2–5 years with CD4^+^T cell counts below 200 cells/µL and a plasma viral load below 50 copies/mL (within one month before baseline). Exclusion criteria included prior use of fusion inhibitors targeting gp41, opportunistic infections or cancer, and abnormalities such as low HGB (<90 g/L), low WBC (<3.0×10^9^/L), low NEUT (<1×10^9^/L), low PLT (<75×10^9^/L), high SCr (>1.5 ULN), elevated AST/ALT/AKP (>3 ULN), and elevated TBIL (>2 ULN). Ineligible participants included pregnant or lactating women, drug abuse cases, and individuals with severe mental or neurological disorders, severe gastrointestinal ulcers, acute hepatitis, and pancreatitis. Contraceptive measures were required for female participants during the study period.

### Participants in the laboratory analysis

**Figure 1:**
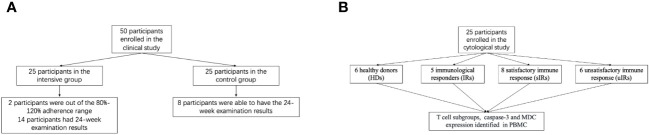
**(A)** Flow diagram showing the sample population enrolled in the clinical study. **(B)** Flow diagram showing the sample population enrolled in the cytological study.

Peripheral blood samples were only collected from those who were willing to take additional sampling, including 6 uninfected healthy donors (HDs); 5 immunological responders (IRs, immune reconstitution after ART initiation); 8 satisfactory immune response (sIRs, previous incomplete immune reconstitution and had CD4^+^T cell counts >200 cells/µL after ABT); 6 unsatisfactory immune response (uIRs, previous incomplete immune reconstitution and had CD4^+^T cell counts <200 cells/µL after receiving regimens including ABT). sIRs and uIRs collected peripheral blood samples within 3 months after ABT administration. At the time of comparison, the sample size of four groups was sufficient to be statistically significant. Peripheral blood samples were obtained to identify T cell subgroups, and caspase-3 and MDC expression in PBMC (**Figure 1B**). All patients were enrolled after written informed consents were obtained.

### Endpoints and assessments

The primary outcome was the absolute change in CD4^+^T cell counts from baseline to week 12. Secondary outcomes included the absolute change in CD4^+^T cell counts from baseline to week 24, the percentage of patients achieving a CD4^+^T cell counts increase of ≥100 cells/μL or a 30% increase from baseline to week 12, and the change in CD4^+^/CD8^+^ cell ratio from baseline to week 12.

Safety indexes were the occurrence rates of adverse events (AEs) and serious adverse events (SAEs) throughout the study period. Adverse events included those related to the study medication, all instances of serious adverse events, and any abnormal laboratory finding recorded during the study period.

### Peripheral blood mononuclear cell collection

PBMC were obtained from EDTA anticoagulant peripheral blood samples by Ficoll-Paque gradient centrifugation (Amersham Pharmacia Biotech, Sweden). All samples were analyzed within 4 h of collection.

### Immunofluorescence staining and flow cytometry

PBMC were incubated with conjugated antibodies for 30 min at 4°C. Antibodies were anti-human CD38-APC, CD3-FITC, CD45RA-PE, CD8-APC-CY7, CCR7-BV421, and CD31-BV510 (BD Biosciences, San Diego, CA, USA). Data acquisition was carried out on a Canto II flow cytometer (BD Bioscience) and data were analyzed with the FlowJo 10.0 Software (Tree Star, Ashland, OR, USA)

### Autophagy assays

RPMI-1640 medium (GIBCO, Grand Island, NY, USA) containing 10% FBS and 80μl MDC (Monodansylcadaverine) (10 mg/ml in DMSO, Sigma-Aldrich, USA) was used for culturing PBMC at 3*10^5^ in each well of a 6-well plate. After 4 h of culture at 37°C with 5% CO_2_, some cells growing on glass slides were washed with 2 mL phosphate-buffered saline (PBS), and 20μl Antifade Mounting Medium with DAPI was added prior to fluorescence microscopy. The remaining cells were washed with 2 mL PBS twice and incubated for 30 min with conjugated antibodies, i.e., anti-human CD3-BV421, CD45RA-PE, CD8-APC-CY7, CD4-PE-CY7 and CD31-BV510. Data acquisition was performed on Canto II flow cytometry (BD Bioscience).

### Apoptosis assays

PBMC were surface-stained with anti-human CD38-APC, CD3-BV421, CD45RA-PE, CD4-PE-CY7, CD8-APC-CY7, and CD31-BV510 for 30 min at 4°C. After addition of the fixation/permeabilization solution (BD, USA) according the manufacturer’s instructions, intracellular staining was performed with caspase-3-APC antibodies (BD Biosciences, San Diego, CA, USA) for 30 min at 4°C.

### Statistical analysis

Continuous data with normal distribution were expressed as mean and standard deviation (SD), while those with skewed distribution were presented as median and interquartile range (IQR). The treatment and control groups were compared by the t-test and Wilcoxon test for continuous variables; the Chi-squared test and Fisher’s exact test were used for categorical measures. General Estimation Equation (GEE) was applied for the analysis of longitudinal data, including changes in indexes from baseline at various follow-up times and their potential influential factors. All statistical analyses used R Studio (version 4.2.1). P<0.05 was considered statistically significant.

In the laboratory analysis, the unpaired two-tailed Student’s t-test was used to compare normally distributed data, with the Mann-Whitney U or Wilcoxon matched-pairs signed-rank test used for unpaired and paired data. The Kruskal-Wallis test followed by Dunn’s multiple comparison was used for two or more independent samples. In this study, SPSS version 21 (SPSS, Chicago, IL, USA) and GraphPad Prism 10 (GraphPad Software, San Diego, CA, USA) were employed for statistical analysis. The Chi-square test was utilized to compare categorical variables.

## Results

Totally 50 INRs enrolled from November 2021 to May 2023 were assigned to the intensive and control groups at a ratio of 1:1 based on the participants’ preference as ABT requires weekly intravenous administration (**Figure 1A**). **Figure 1A** depicts the enrollment process and the sample sizes included in various analyses. Demographic and baseline features were generally balanced between the two groups (**Tables 1**, **2**).

**Table 1:** Baseline characteristics of the participants in the clinical study.

Characteristic	Intensive group N=25	Control group N=25	P-value
Male, n (%)	24 (96.0)	24 (96.0)	1.000
ART regimens, n (%)			
2 NRTIS+NNRTIS	5 (20.0)	9 (36.0)	0.452
2 NRTIS+PIS	3 (12.0)	4 (16.0)	
2NRTIS +INSTIS	17 (68.0)	12 (48.0)	
Age^#^ (years)	48.00 (42, 56)	53.00 (38, 57)	0.954
ART duration* (years)	7.46 (2.91)	7.73 (2.82)	0.744
Baseline laboratory data			
CD4 count* (cells/µL)	287 (99)	280(105)	0.813
CD8 count^#^ (cells/µL)	577 (412, 907)	650 (572, 910)	0.200
CD4/CD8 ratio^#^	0.45 (0.31, 0.65)	0.44 (0.28, 0.55)	0.357

#Value represents the median (IQR) * Value represents the mean (SD)

**Table 2:** Outcomes of treatment efficacy parameters at Week 12 & Week 24.

Treatment efficacy parameter	Intensive group (n=25)		Control group (n=25)	P-value
CD4^+^T absolute counts cells/µL				
Baseline*	287 (99)		280(105)	0.813
Week 12*	356 (133)		282 (110)	0.037
Change in CD4^+^T cells at week 12 from baseline^#^	45 (24, 122)		-5(-23, 13)	<0.001
CD8^+^T absolute counts cells /µL				
Baseline^#^	576 (412, 907)		650 (572, 910)	0.2
Week 12^#^	690 (567, 919)		608 (411, 1095)	0.528
Change in CD8^+^T cells at week 12 from baseline^#^	82 (18, 158)		-41 (-137, 207)	0.043
CD4/CD8 ratio				
Baseline^#^	0.45 (0.31, 0.65)		0.44 (0.28, 0.55)	0.357
Week 12^#^	0.44 (0.32, 0.63)		0.44 (0.30, 0.53)	0.541
Change in CD4/CD8 ratio at week 12 from baseline^#^	0.01 (-0.04, 0.07)		0.01 (-0.02,0.09)	0.455
**Proportion of participants with CD4^+^T cell count increase ≥100 cells/µL or ≥30% from baseline at week 12**	10 (40.0)		2 (8.0)	0.018
Treatment efficacy parameter	Discontinued ABT after Week 12 (n=6)	Continued ABT after Week 12 (n=8)	Control group (n=25)	P-value
CD4^+^T absolute counts cells/µL				
Baseline*	294 (96)	254 (100)	280 (105)	0.748
Week 24*	374 (190)	331 (161)	259 (141)	0.415
Change in CD4^+^T cells at week 24 from baseline^#^	47 (25, 81)	56 (36, 122)	-5 (-16, 11)	0.039
Change in CD4^+^T cells at week 24 from week 12*	-21 (92)	-12 (32)	-4 (21)	0.826
CD8^+^T absolute counts cells /µL				
Baseline^#^	474 (401, 918)	408 (345, 589)	650 (572, 910)	0.059
Week 24^#^	643 (534, 914)	540 (420, 608)	587 (519, 630)	0.609
Change in CD8^+^T cells at week 24 from baseline^#^	74 (-43, 125)	82 (-2, 193)	-28 (-130, 137)	0.468
Change in CD8^+^T cells at week 24 from week 12^#^	-226 (-478, 90)	-61 (-111, -24)	-19 (-69, 38)	0.587
CD4/CD8 ratio				
Baseline^#^	0.57 (0.34, 0.78)	0.47 (0.43, 0.63)	0.44 (0.28, 0.55)	0.326
Week 24^#^	0.54 (0.36, 0.74)	0.53 (0.45, 0.71)	0.37 (0.28, 0.49)	0.303
Change in CD4/CD8 ratio at week 24 from baseline^#^	0.01 (-0.04, 0.07)	-0.02 (-0.04, 0.07)	0.00 (-0.02, 0.05)	0.978
Change in CD4/CD8 ratio at week 24 from week 12*	0.03 (0.12)	0.00 (0.14)	0.01 (0.12)	0.947
**Proportion of participants with CD4^+^T cell count increase ≥100 cells/µL or ≥30% at week 24**	2 (33.3)	4 (50.0)	0 (0.0)	<0.001

The clinical and sample data for 25 participants in the laboratory analysis are found in the **Supplementary Materials** (**Supplementary Table S3**).

### CD4^+^T cell counts were increased by ABT administration

**Figure 2:**
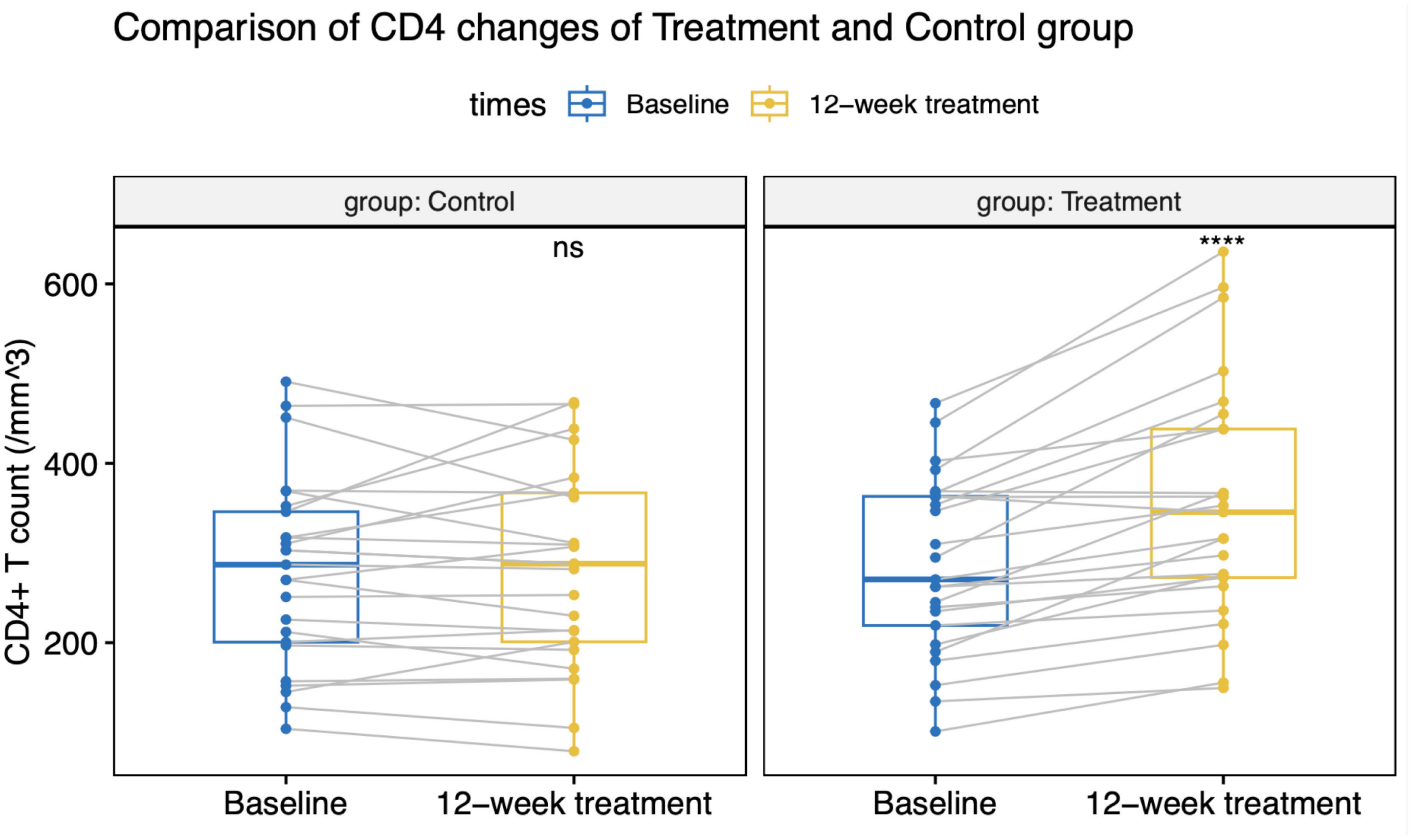
CD4^+^T cell count changes in the treatment and control groups.

**Figure 3:**
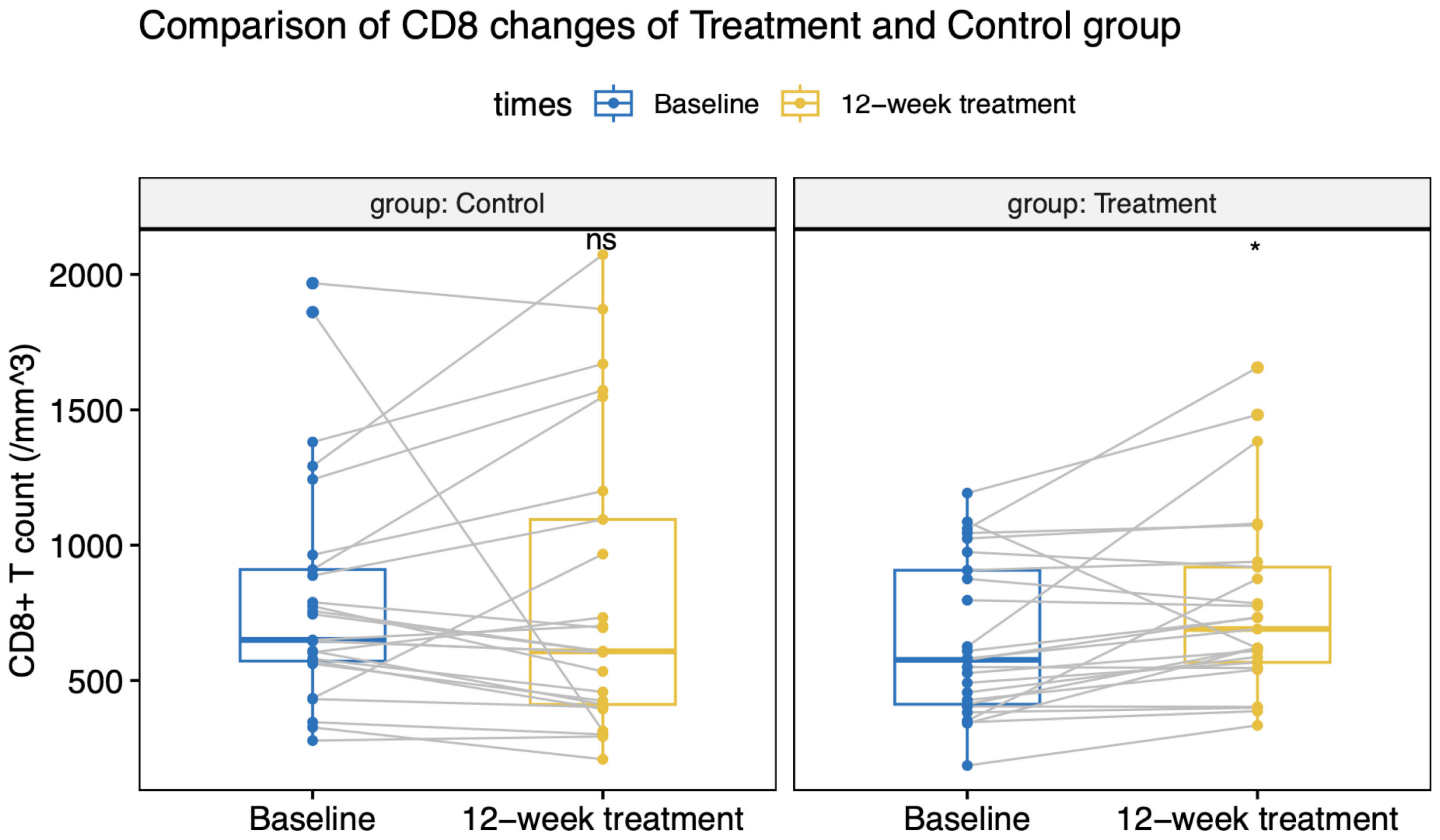
CD8^+^T cell count changes in the treatment and control groups.

All patients maintained virologic suppression during the study period. CD4^+^T cell counts at week 12 was 356 (133) cells/µL in the intensive group, versus 282 (110) cells/µL in the control group. Only the intensive group showed a statistically significant CD4^+^ and CD8^+^ cell count increase from the baseline value (**Figures 2**, **3**). CD4^+^T cell counts at week 12 increased significantly in the intensive group compared to the control group (45 vs. -5 cells/µL, *p*<0.001; **Table 2**).

At week 12, 40% of participants in the intensive group had a CD4^+^T cell count increase over 100 cells/µL (or >30% increase from baseline), whereas only 8% of patients in the control group had a CD4^+^T cell count increase (*p*=0.018; **Table 2**). Following a 12-week ABT administration, 11 participants were loss to follow-up. 6 participants discontinued ABT and 8 participants continued ABT at their discretion and were continuously observed for up to 24 weeks. By the 24^th^ week, CD4^+^T cell counts in the intensive group showed a slight decline from week 12, but still notably higher than baseline values (**Table 2**).

### Participants over 45 yrs. or ART treatment history over 5 yrs. benefit more from ABT

Effectiveness was assessed after stratification by age and ART treatment history. Both the intensive and control groups consisted of 17 patients aged over 45 years.

In the intensive group, patients aged over 45 years had a notably higher increase in CD4^+^T cell counts at 12 weeks (50 vs. -13 cells/µL, *p*= 0.001; **Supplementary Table S1**). Furthermore, the effectiveness of ABT was particularly evident in patients administered ART for more than 5 years with baseline CD4+T cell counts below 500 cells/µL (**Supplementary Table S2**).

### CD4/CD8 ratio were comparable between two groups

CD4/CD8 ratio changes at week 12 from baseline in both study groups were comparable with no significant difference [intensive group vs. control group: 0.01 (-0.04, 0.07) vs. 0.01 (-0.02, 0.09), *p*=0.455].

### Participants were well tolerated with ABT treatment

During the 12 weeks of ABT treatment, no participants experienced injection site reactions, and no clinical adverse events related to ABT were detected. Additionally, there were no self-reported complaints during the treatment period. In the laboratory analysis, the commonest abnormality was elevated triglyceride after 12 weeks of ABT intensive therapy (16%) (**Table 3**). All hematologic and biochemical abnormalities were manageable. No ABT-associated treatment discontinuation was performed, and there were no drug-related SAEs.

**Table 3:** Incidence rates of laboratory parameter abnormalities in both groups after 12 weeks of ABT treatment.

	Intensive group (N=25)	Control group (N=25)
	≥1 grade abnormalities N (%)	≥1 grade abnormalities N (%)
Hyperuricemia	1/25 (4%)	/
Hyperglycemia	1/25 (4%)	1/25 (4%)
Increased total bilirubin	1/25 (4%)	/
Increased cholesterol	1/25 (4%)	/
Increased triglyceride	4/25 (16%)	/

### Naïve CD4^+^T cells were decreased in PLWHA with INR

**Figure 4:**
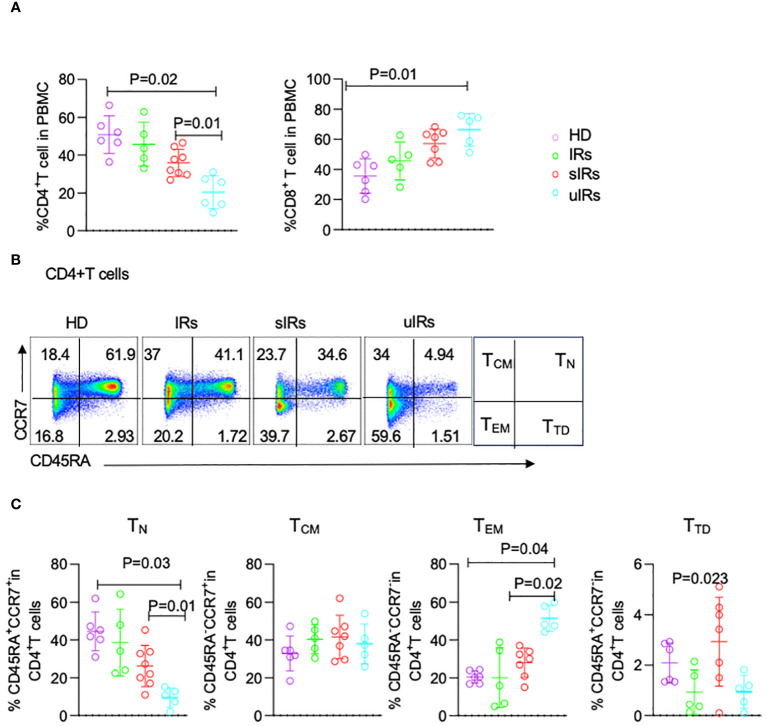
Flow cytometry analysis of CD4^+^T cell subsets in PBMC from HDs, IRs, sIRs and uIRs. **(A)** Percentages of CD4^+^T cells (left) and CD8^+^T cells (right) in PBMC for the four groups. **(B)** Ideograph (right) and scatter diagram of flow cytometry analysis of human CD4^+^T cells classified by CD45RA and CCR7 expression. According to the expression patterns of CD45RA and CCR7, CD4^+^ T cells were divided into naïve T cells (T_N_), central memory T cells (T_CM_), effector memory T cells (T_EM_) and terminal differentiation cells (T_TD_). **(C)** Percentages of T_N_, T_CM_, T_TD_ and T_EM_ in PBMC for the four groups. HDs, healthy donors; uIRs, participants with unsatisfactory immune response to ABT; sIRs, participants with satisfactory immune response to ABT, IRs, immunological responders.

As shown in **Figure 4A**, compared with HDs and PLWHA with IR and sIR, PLWHA with uIR displayed significantly decreased percentages of CD4^+^T cells in PBMC (50.8%, 45%, 35.9% vs 20%; *p*<0.05). Meanwhile, significant increases in CD8^+^T cell proportions were observed in uIRs, sIRs and IRs compared with the HD group (35.5%, 45.6%, 57.1% vs 66.3%; p<0.01) (**Figure 4A**).

To explore the mechanism of CD4^+^T cell elevation by ABT in INRs, the percentages of CD4^+^T cells were assessed in the four groups. The proportion of naïve T (T_N_) cells was significantly lower in PLWHA with uIR compared with the HD group (9.54% vs 44.5, 9.45% vs 44.5% p=0.03). Meanwhile, T_N_ cells were also significantly decreased in PLWHA with uIR than in those with sIR (9.54% vs 26.2%, *p*=0.01) (**Figures 4B, C**). The percentage of effector memory T (TEM) cells was higher in PLWHA with uIR compared with HDs and PLWHA with sIR (51.5%, 20.5% vs 28.4%, p=0.001). The proportion of terminally differentiated T (T_TD_) cells was highest in participants with sIR. The amounts of central memory T (T_cm_) cells were comparable in the four groups (**Figure 4C**).

### CD4^+^ T thymocyte output was decreased in the INR group

**Figure 5:**
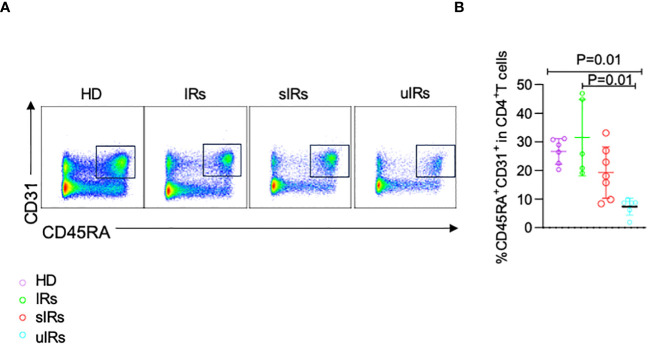
Flow cytometry analysis of CD45RA^+^CD31^+^CD4^+^ T cells in PBMC samples from the four different populations. **(A)** Representative plots of flow cytometry analysis with cells gated by CD45RA and CD31 expression. **(B)** Percentages of CD45RA^+^CD31^+^ cells among CD4^+^T cells in PBMC samples from the four groups.

To determine the cause of the deficiency in naïve CD4^+^T cells, CD45RA^+^CD31^+^ cells were detected among CD4^+^T cells, which reflect thymocyte migration (Iio et al., 2023). Compared with HDs and PLWHA with sIR, decreased proportion of CD45RA^+^CD31^+^CD4^+^ T cells was found in PLWHA with uIR (26.5%, 31.5%, 19.3% vs 7.4%, *p*=0.001) (**Figures 5A, B**), indicating lower thymocyte output might account for T_N_ deficiency in PLWHA with uIR.

### Increased apoptosis of naïve CD4^+^T cells in the INR group

**Figure 6:**
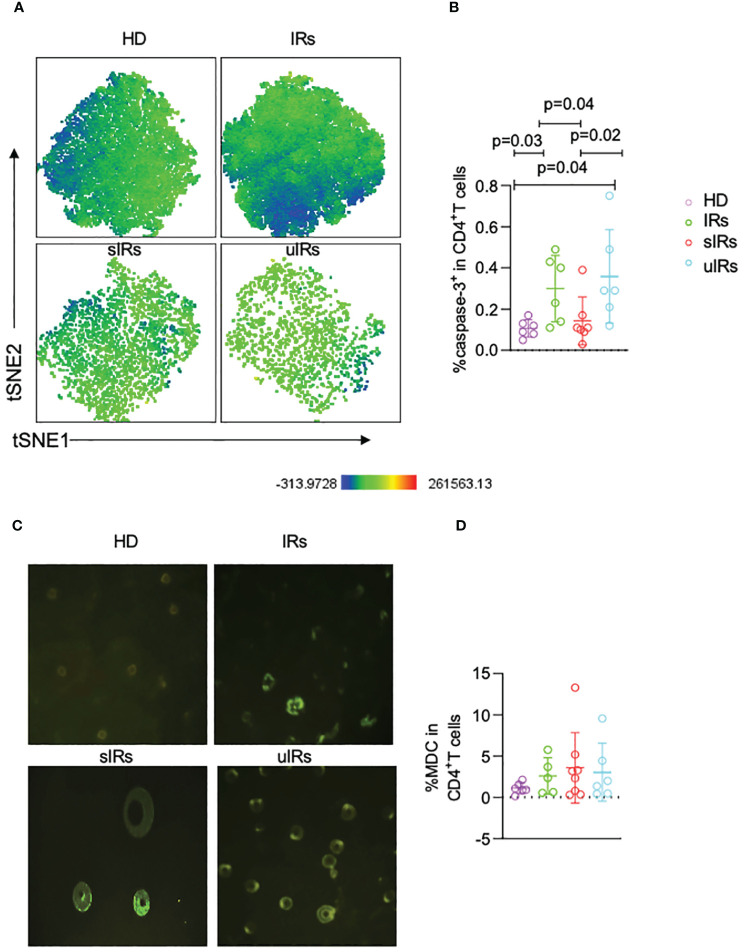
Caspase-3 and MDC expression levels in CD4^+^T cells assessed by flow cytometry and fluorescence microscopy in the four different populations. **(A)** Representative t-SNE (t-distributed stochastic neighbor embedding) plots of caspase-3 expression in CD45RA^+^CD31^+^CD4^+^ T cells. **(B)** Percentages of caspase 3^+^ cells in CD45RA^+^CD31^+^CD4^+^ T cells in PBMC samples from the four different populations. **(C)** Representative micrographs obtained by fluorescence microscopy of PBMC in the four groups. **(D)** Percentages of MDC^+^CD4^+^T cells in PBMC in the four different populations, obtained by flow cytometry.

To further investigate the roles of apoptosis and autophagy in immune reconstitution, the expression of capspase-3 on CD4^+^T cells was examined. The percentages of caspase 3^+^CD45RA^+^CD31^+^ cells were significantly lower in participants with sIR after ABT compared with participants with uIR and IR (*p*=0.04) (**Figures 6A, B**). However, there were comparable MDC levels in the four groups (**Figures 6C, D**).

## Discussion

Despite the significant increase in life expectancy for PLWHA worldwide due to antiretroviral therapy (ART), higher rates of AIDS-related or non-AIDS related diseases and death were found in patients with CD4^+^T cell counts below 200 cells/μl due to various factors such as reduced thymic output, dysfunction in bone marrow hematopoiesis, ongoing viral replication, immune exhaustion, and abnormal immune activation (Kelley et al., 2009; Li et al., 2011). This study revealed that ABT can remarkably help immune reconstitution by avoiding T_N_ cell depletion and reducing cell apoptosis through clinical and cytological results.

CD4^+^T cell count is the dominant indicator evaluating the immune reconstitution in PLWHA. In this study, a three-month intensification ART with ABT treatment significantly enhanced the recovery of CD4^+^T cells by 45 cells/μl in INRs and the treatment could uphold the increase in CD4^+^T cell count till week 24 which had not being achieved by other ARV intensification regimens in previous studies (Corbeau and Reynes, 2011).

Though elevated CD4^+^T cell counts can be realized among most participants with ABT intensified ART, some participants still could not achieve immune reconstruction (CD4^+^T cell count <200 cells/μl), which may result from inadequate duration of ABT use, late ART onset and/or poor treatment compliance.

From a theoretical perspective, both apoptosis and pyroptosis contribute to CD4^+^T cell death, and gp41 and Env fusion is essential for apoptosis in bystander CD4^+^T cells (Doitsh et al., 2014). It was also reported that the fusion inhibitors T20 and C34 block Env-mediated autophagy (Meissner et al., 2006; Denizot et al., 2008). ABT is a chemically modified peptide designed to target the HIV-1 envelope protein gp41and blocks HIV entry into cells which can thus inhibit cell apoptosis and autophagy (Garg and Blumenthal, 2008). ABT can work for patients under viral suppression as gp41 can still be detected in patients with HIV-1 RNA content under the lower limit (Delagreverie et al., 2019). In sIRs, the proportion of apoptotic CD4^+^T cells marked by capspase-3 was reduced, suggesting that ABT may play a role in immune reconstitution by decreasing cell apoptosis.

Naive CD4^+^T cells serve as a crucial reservoir supplying the central memory. Therefore, the overall reconstitution of CD4^+^T cells depends on the integrity of the naive CD4^+^T cell population in lymphoid tissues (Li et al., 2011). Avoiding the depletion of T_N_ cells may contribute to improving immune reconstitution. In uIRs, the proportion of T cells and T_N_ cells were significantly lower, while that of T_EM_ cells was notably higher. Previous study with the kinetics of different T cell subsets also showed that less naive CD4^+^T cell increase in the INR group than in the IR group and the change of the T cells is primarily attributed to the change of T_N_ cells (Li et al., 2011). The observed reduction in T_EM_ cells indicates a potential decline in the number of HIV RNA-expressing cells and a decrease in latent HIV reservoirs, which is one of the major factors contributing to the increase of CD4^+^T cells, thereby contributing immune reconstitution (Iio et al., 2023). The cytological study results showed that ABT may help INRs achieve immune recovery, particularly by helping avoid T_N_ cell depletion. Elderly participants are more immunocompromised with poor output of thymic immune function (Palmer, 2013). Among all participants, those over 45 years showed the most prominent response to ABT which was consistent with results from (5R)-5-hydroxytriptolide phase II study (Cao et al., 2023). Therefore, PLWHA with T_N_ cell depletion over 45 years are more likely to benefit from the ABT intensive regimen.

According to a recent study, thymic output dysfunction is a crucial factor affecting the failure of immune reconstitution in patients with incomplete immune reconstitution (Li et al., 2011). CD45RA^+^CD31^+^ cells as an indicator of recent thymic emigrants were significantly decreased in individuals with uIR, indicating reduced thymic output function (Iio et al., 2023).

This study had several limitations. Firstly, analyzing the long-term effects of ABT intensification on immune recovery was challenging as only half of the patients had 24-week data. Individuals with better outcomes are more likely to have 24-week data and bias towards the intensive group. Secondly, the laboratory analysis was a cross-sectional investigation, and a prospective study with a larger sample is required. Thirdly, in the clinical study, participants were not randomized into the two arms, which may lead to selection bias. Fourthly, fluorescence microscopy cannot distinguish whether the MDC expression found in PBMC originates from CD4^+^T cells.

**Figure 7:**
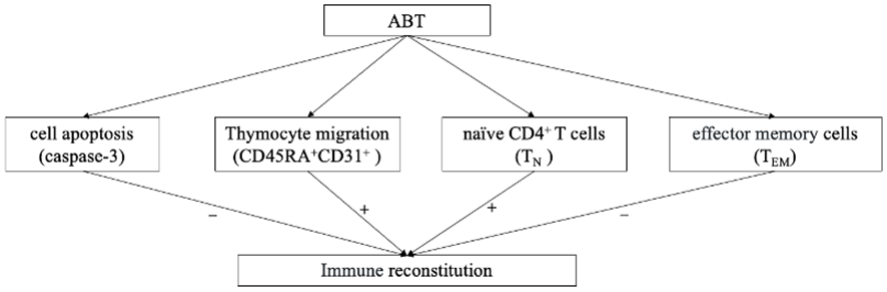
The logical flowchart of cytological results. “+” indicate enhanced by ABT; “-” indicate diminished by ABT.

In conclusion, ABT enhances CD4^+^T cell recovery in PLWHA with INRs. Enhancing thymocyte cell amounts, decreasing HIV reservoir replication and reducing naïve CD4^+^T cell apoptosis might be the main immune reconstitution mechanisms of ABT (**Figure 7**). Intensive ABT treatment might be a promising therapeutic option for managing INRs, in addition to the standard ART.

## Data availability statement

The original contributions presented in the study are included in the article/**Supplementary Material**. Further inquiries can be directed to the corresponding author/s.

## Ethics statement

The studies involving humans were approved by Medical Ethics Committee of Tianjin Second People’s Hospital. The studies were conducted in accordance with the local legislation and institutional requirements. The participants provided their written informed consent to participate in this study. The animal study was approved by Medical Ethics Committee of Tianjin Second People’s Hospital. The study was conducted in accordance with the local legislation and institutional requirements. Written informed consent was obtained from the individual(s) for the publication of any potentially identifiable images or data included in this article.

## Author contributions

LF: Writing – original draft, Software, Project administration, Methodology, Formal analysis, Data curation, Writing – review & editing, Supervision, Investigation. PM: Resources, Conceptualization, Writing – review & editing, Supervision, Investigation. YH: Writing – review & editing, Project administration, Data curation. RL: Writing – review & editing, Project administration. JD: Writing – review & editing, Project administration, Methodology. SY: Writing – review & editing, Writing – original draft, Software, Formal analysis, Data curation. YuL: Writing – review & editing, Writing – original draft, Supervision, Formal analysis, Data curation. DX: Writing – review & editing, Supervision, Formal analysis, Conceptualization. MH: Writing – review & editing, Supervision, Conceptualization. RS: Writing – review & editing, Software, Project administration, Data curation. YaL: Writing – review & editing, Software, Project administration, Investigation. AY: Writing – review & editing, Supervision, Investigation. QZ: Writing – review & editing, Supervision, Formal analysis.

## References

[B1] Cao W. Liu X. Han Y. Song X. Lu L. Li X.. (2023). (5R)-5-hydroxytriptolide for HIV immunological non-responders receiving ART: a randomized, double-blinded, placebo-controlled phase II study. Lancet regional Health Western Pacific 34, 100724. doi: 10.1016/j.lanwpc.2023.100724 37283977 PMC10240372

[B2] Chen L. Dai H. Wang J. Wang M. Ma Y. Ma F.. (2022). Comparing albuvirtide plus ritonavir-boosted lopinavir regimen to two nucleotide reverse transcriptase inhibitors plus ritonavir-boosted lopinavir in HIV-infected individuals who failed initial treatment: a retrospective comparative cohort study. Ann. Trans. Med. 10, 1125. doi: 10.21037/atm PMC9652537 36388832

[B3] Corbeau P. Reynes J. (2011). Immune reconstitution under antiretroviral therapy: the new challenge in HIV-1 infection. Blood 117, 5582–5590. doi: 10.1182/blood-2010-12-322453 21403129

[B4] Delagreverie H. M. Grude M. Lambert-Niclot S. Nere M. L. Jadand C. Leport C.. (2019). Anti-gp41 antibody levels reflect HIV viral suppression and cellular reservoir in long-term antiretroviral-treated trial participants. J. antimicrobial chemotherapy 74, 1389–1394. doi: 10.1093/jac/dkz004 30690509

[B5] Denizot M. Varbanov M. Espert L. Robert-Hebmann V. Sagnier S. Garcia E.. (2008). HIV-1 gp41 fusogenic function triggers autophagy in uninfected cells. Autophagy 4, 998–1008. doi: 10.4161/auto.6880 18818518

[B6] Doitsh G. Galloway N. L. Geng X. Yang Z. Monroe K. M. Zepeda O.. (2014). Cell death by pyroptosis drives CD4 T-cell depletion in HIV-1 infection. Nature 505, 509–514. doi: 10.1038/nature12940 24356306 PMC4047036

[B7] Engsig F. N. Zangerle R. Katsarou O. Dabis F. Reiss P. Gill J.. (2014). Long-term mortality in HIV-positive individuals virally suppressed for >3 years with incomplete CD4 recovery. Clin. Infect. diseases: an Off. Publ. Infect. Dis. Soc. America 58, 1312–1321. doi: 10.1093/cid/ciu038 PMC6276895 24457342

[B8] Garg H. Blumenthal R. (2008). Role of HIV Gp41 mediated fusion/hemifusion in bystander apoptosis. Cell. Mol. Life sciences: CMLS 65, 3134–3144. doi: 10.1007/s00018-008-8147-6 PMC2574860 18500445

[B9] Iio K. Kabata D. Iio R. Shibamoto S. Watanabe Y. Morita M.. (2023). Decreased thymic output predicts progression of chronic kidney disease. Immun. ageing: I A 20, 8. doi: 10.1186/s12979-023-00333-z 36788556 PMC9926722

[B10] Kelley C. F. Kitchen C. M. Hunt P. W. Rodriguez B. Hecht F. M. Kitahata M.. (2009). Incomplete peripheral CD4+ cell count restoration in HIV-infected patients receiving long-term antiretroviral treatment. Clin. Infect. diseases: an Off. Publ. Infect. Dis. Soc. America 48, 787–794. doi: 10.1086/597093 PMC2720023 19193107

[B11] Li T. Wu N. Dai Y. Qiu Z. Han Y. Xie J.. (2011). Reduced thymic output is a major mechanism of immune reconstitution failure in HIV-infected patients after long-term antiretroviral therapy. Clin. Infect. diseases: an Off. Publ. Infect. Dis. Soc. America 53, 944–951. doi: 10.1093/cid/cir552 21960716

[B12] Medicine A-ABoCAoC (2020). Consensus of integrative medicine treatment experts on poor reconstruction of HIV immune function. Acta Chin. Med. 35, 281–284. doi: 10.16368/j.issn.1674-8999.2020.02.064

[B13] Meissner E. G. Zhang L. Jiang S. Su L. (2006). Fusion-induced apoptosis contributes to thymocyte depletion by a pathogenic human immunodeficiency virus type 1 envelope in the human thymus. J. Virol. 80, 11019–11030. doi: 10.1128/JVI.01382-06 16956934 PMC1642149

[B14] Noiman A. Esber A. Wang X. Bahemana E. Adamu Y. Iroezindu M.. (2022). Clinical factors and outcomes associated with immune non-response among virally suppressed adults with HIV from Africa and the United States. Sci. Rep. 12, 1196. doi: 10.1038/s41598-022-04866-z 35075147 PMC8786968

[B15] Palmer D. B. (2013). The effect of age on thymic function. Front. Immunol. 4, 316. doi: 10.3389/fimmu.2013.00316 24109481 PMC3791471

[B16] Rb-Silva R. Goios A. Kelly C. Teixeira P. João C. Horta A.. (2019). Definition of immunological nonresponse to antiretroviral therapy: A systematic review. J. acquired Immune deficiency syndromes (1999) 82, 452–461. doi: 10.1097/QAI.0000000000002157 31592836

[B17] Su B. Yao C. Zhao Q. X. Cai W. P. Wang M. Lu H. Z.. (2020). Efficacy and safety of the long-acting fusion inhibitor albuvirtide in antiretroviral-experienced adults with human immunodeficiency virus-1: interim analysis of the randomized, controlled, phase 3, non-inferiority TALENT study. Chin. Med. J. 133, 2919–2927. doi: 10.1097/CM9.0000000000001273 33252379 PMC7752691

[B18] Su B. Yao C. Zhao Q. X. Cai W. P. Wang M. Lu H. Z.. (2022). Long-acting HIV fusion inhibitor albuvirtide combined with ritonavir-boosted lopinavir for HIV-1-infected patients after failing the first-line antiretroviral therapy: 48-week randomized, controlled, phase 3 non-inferiority TALENT study. J. infection 85, 334–363. doi: 10.1016/j.jinf.2022.05.034 35659547

[B19] Takuva S. Maskew M. Brennan A. T. Long L. Sanne I. Fox M. P. (2014). Poor CD4 recovery and risk of subsequent progression to AIDS or death despite viral suppression in a South African cohort. J. Int. AIDS Soc. 17, 18651. doi: 10.7448/IAS.17.1.18651 24594114 PMC3942566

[B20] Tang W. Song X. Y. Cao J. Liu C. Zheng F. (2023). Rescue therapy with an albuvirtide-based antiretroviral regimen in an HIV-infected child with multidrug resistance and multiple opportunistic infections: a case report. AIDS Res. Ther. 20, 60. doi: 10.1186/s12981-023-00560-w 37641133 PMC10463494

[B21] Yang X. Su B. Zhang X. Liu Y. Wu H. Zhang T. (2020). Incomplete immune reconstitution in HIV/AIDS patients on antiretroviral therapy: Challenges of immunological non-responders. J. leukocyte Biol. 107, 597–612. doi: 10.1002/JLB.4MR1019-189R 31965635 PMC7187275

[B22] Zhang H. Jin R. Yao C. Zhang T. Wang M. Xia W.. (2016). Combination of long-acting HIV fusion inhibitor albuvirtide and LPV/r showed potent efficacy in HIV-1 patients. AIDS Res. Ther. 13, 8. doi: 10.1186/s12981-016-0091-1 26865854 PMC4748529

