# Supplementary material for: Enhanced immune reconstitution with albuvirtide in HIV-infected immunological non-responders
Source: Front Cell Infect Microbiol. 2024 Jun 21;14:1397743. doi: 10.3389/fcimb.2024.1397743 (PMC11227255; doi:10.3389/fcimb.2024.1397743)
Supplement: Supplementary file 1 [file Table_1.docx]

**Supplementary Table S1. Efficacy analyses stratified by age.**

| Efficacy outcome | Intensive group | Control group | *P*-value |
| --- | --- | --- | --- |
| Patients with age ≤45 years | | | |
| No. of patients | 9 | 8 |  |
| Change in CD4^+^ cell count at week 12, cells/mm^3^ | | | |
| Mean | 38 [17, 91] | 0 [-5, 24] | 0.043 |
| Change in CD4^+^ cell count at week 24, cells/mm^3^ | | | |
| Mean | 88 [63, 117] | 11 [-22, 43] | 0.245 |
| Patients with age >45 years | | | |
| No. of patients | 16 | 17 |  |
| Change in CD4^+^ cell count at week 12, cells/mm^3^ | | | |
| Mean | 50 [32,123] | -13 [-25,13] | 0.001 |
| Change in CD4^+^ cell count at week 24, cells/mm^3^ | | | |
| Mean | 42 [18, 60] | -5 [-13, 8] | 0.045 |

**Supplementary Table S2. Efficacy analyses, stratified by ART treatment duration.**

| Efficacy outcome | Intensive group | Control group | *P*-value |
| --- | --- | --- | --- |
| Patients with ART > 5 years | | | |
| No. Of patients | 20 | 22 |  |
| Change in CD4^+^ cell count at week 12 | | | |
| Mean | 62 [33, 127] | -7 [-25, 31] | <0.001 |
| Change in CD4^+^ cell count at week 24 | | | |
| Mean | 63 [41, 128] | -12 [-19, 12] | 0.01 |
| Patients with ART < 5 years and > 2 years | | | |
| No. Of patients | 4 | 3 |  |
| Change in CD4^+^ cell count at week 12 | | | |
| Mean | 25 [11, 38] | 3 [2, 5] | 0.289 |
| Change in CD4^+^ cell count at week 24 | | | |
| Mean | 2 [-6, 10] | 1 [1, 1] | 1 |

**Supplementary Table S3: Characteristics of participants in the laboratory analysis**

|  | HDs (n=6) | IRs (n=5) | SIRs (n=8) | INRs (n=6) |
| --- | --- | --- | --- | --- |
| Male, n (%) | 6 (100%) | 5 (100%) | 8 (100%) | 6 (100%) |
| Age [mean (sd)] | 41.83 (12.07) | 42.2 (5.97) | 53.5 (11.22) | 55.2 (9.04) |
| ART duration [mean (sd)] | / | 7.12 (2.75) | 7.11 (1.78) | 4.07 (1.71) |
| Current ART regimens, n (%) | / | INSTIs+NRTIs 2 (40%)  2NRTIs+PIs 2 (40%)  INSTIs 1 (20%) | INSTIs+NRTIs 7 (87.5%)  2NRTIs+NNRTIs 1 (12.5%) | INSTIs+NRTIs 6 (100%) |
